# Immediate Effects of Sprinter-Pattern Exercise on the Lordotic Curve and Abdominal Muscle Activity in Individuals with Hyperlordosis

**DOI:** 10.3390/medicina59122177

**Published:** 2023-12-15

**Authors:** Sangbong Lee, Hyunjoong Kim, Jihye Jung, Seungwon Lee

**Affiliations:** 1 Department of Physical Therapy, Graduate School of Sahmyook University, 815, Hwarang-ro, Nowon-gu, Seoul 01795, Republic of Korea; leesb109109@gmail.com; 2 Neuromusculoskeletal Science Laboratory, 15, Gangnam-daero 84-gil, Seoul 06232, Republic of Korea; hyun-joongkim@nmslab.org; 3 Institute of SMART Rehabilitation, Sahmyook University, 815, Hwarang-ro, Nowon-gu, Seoul 01795, Republic of Korea; jihye3752@gmail.com; 4 Department of Physical Therapy, Sahmyook University, 815, Hwarang-ro, Nowon-gu, Seoul 01795, Republic of Korea

**Keywords:** lordosis, abdominal muscles, exercise, electromyography, radiography

## Abstract

*Background and Objectives*: Abdominal muscle exercises with limb movements are more effective for trunk stabilization than traditional exercises involving trunk flexion alone. This study examined the effects of abdominal exercises incorporating sprinter pattern and crunch exercises on changes in the lordotic curve and abdominal muscle activation in individuals with low back pain caused by hyperlordosis resulting from weak abdominal muscles. *Materials and Methods*: In this single-blind, randomized controlled trial, a total of 40 participants with hyperlordosis were recruited and randomly assigned to perform either sprinter-pattern abdominal exercises or crunch exercises. The participants assigned to each group performed three sets of ten abdominal exercises. The lumbar lordotic angle (LLA) and sacrohorizontal angle (SHA) were assessed prior to and following the intervention, whereas abdominal muscle activity was gauged throughout the intervention period. Changes in the LLA and SHA were measured by radiography. Abdominal muscle activity was measured using electromyography. *Results*: The LLA and SHA decreased significantly in both groups (*p* < 0.001), while the sprinter-pattern exercise group showed a statistically significant decrease compared to the crunch exercise group (*p* < 0.001). In the activity of the abdominal muscles, there was no significant difference in the rectus abdominis muscle between the two groups (*p* > 0.005). However, a significant difference between the external and internal oblique muscles was observed, and the activities of both muscles were significantly higher in the sprinter-pattern exercise group than in the crunch exercise group (*p* < 0.005). *Conclusions*: Abdominal exercise using a sprinter pattern may be effective in reducing lumbar lordosis by strengthening the abdominal muscles in patients with hyperlordosis.

## 1. Introduction

Chronic back pain has become a pressing health issue due to factors such as an increased sedentary lifestyles, lack of physical activity, and unawareness of body imbalances [1]. One of the causes of chronic back pain is the shortening of the erector spinae muscles and the weakening of the abdominal muscles, which can result in changes to the curvature of the spine in the lumbar spine [2]. This combination can lead to hyperlordosis, which puts substantial stress on the back, resulting in back pain [2,3,4].

A deficiency in the abdominal muscles causes hyperlordosis, which leads to a deviation from the normal alignment of the spine, pelvis, and sacrum, resulting in instability in the lumbar segment [5,6,7]. Hyperlordosis can induce pain by reducing the space in the intervertebral foramen [4] and misshaping the spine’s natural curve [2].

Abdominal muscle weakness is commonly found in patients with back pain [3]. Strengthening the abdominal muscles is known to be essential for the spine to maintain neutrality and to restore segmental stability [3]. Therefore, strong abdominal muscles are crucial not only for individuals with back instability, but also for those who are susceptible to developing back pain, as these muscles play an important role in preventing back pain by increasing the space in the intervertebral foramen, thus relieving stress on the facet joints resulting from hyperlordosis and maintaining proper postural control [8,9,10].

Although traditional exercises such as sit-ups and curl-ups are widely used to strengthen the abdomen, they can also increase the risk of lower back injuries and potentially lead to degenerative spinal damage owing to increased pressure on the spine [11,12]. To mitigate these risks, it is recommended to engage in stability exercises that integrate the functional movements of the arms and legs to enhance abdominal activity [13]. Building on this knowledge, Dietz [14] designed integrated pattern training, combining proprioceptor neuromuscular facilitation patterns with walking movement patterns, termed sprinter and skater motions.

In particular, the sprinter pattern enhances body stabilization by strengthening the abdominal muscles, and employs coordinated arm and leg movements to stabilize the trunk [15]. This movement activates the rectus abdominis (RA), external oblique (EO), and internal oblique (IO) [16]. This activation is consistent with research emphasizing the significance of exercises that strengthen posture-related muscles for trunk stabilization and activate crucial muscles for spinal alignment [15,16,17].

While extensive research has been conducted on abdominal exercises to examine their effects on hyperlordosis and the activation of abdominal muscles, the current literature lacks conclusive results regarding the specific exercises that effectively reduce lumbar lordosis and increase activation of abdominal muscles. Therefore, this study aimed to assess and compare the effects of abdominal exercises involving sprinter-pattern motions with traditional crunches, focusing on lumbar anterior flexion and abdominal muscle activity in patients with hypertrophic lumbar lordosis with angles exceeding the norm.

## 2. Materials and Methods

### 2.1. Study Design

This single-blind, randomized controlled trial was conducted from 29 October to 24 November 2019, and adhered to the ethical principles outlined in the Declaration of Helsinki. This study followed the reporting guidelines of the Consolidated Standards of Reporting Trials (CONSORT).

### 2.2. Participants and Ethics

A total of 40 participants (20 males, 20 females) between the ages of 20 and 50 who visited the Shinbaro Orthopedic Clinic in Seoul, Republic of Korea for low back pain were enrolled in the study. Study participant recruitment began in October 2019, and all the data were obtained between October 2019 and November 2019. Participants were voluntarily recruited through a clinic’s bulletin board. Participants were assessed for eligibility using inclusion [18,19,20] and exclusion criteria [21,22,23,24]. Subsequently, participants who met the eligibility criteria were provided with detailed written and verbal explanations of the study procedures and objectives. They voluntarily expressed their willingness to participate and signed the informed consent form before the commencement of the study.

The study procedures were registered with the Clinical Research Information Service, affiliated with the World Health Organization’s International Clinical Trials Registry Platform in the Republic of Korea (No. KCT0005232). Prior to initiating the experiment, we obtained ethical approval from the Sahmyook University Ethics Committee (No. 2-7001793-AB-N012019086HR, 29 October 2019).

#### 2.2.1. Inclusion Criteria

- Adults aged 20–50 years;
- Chronic back pain for more than 3 months;
- Lumbar lordotic angle (LLA) > 50°;
- Sacrohorizontal angle (SHA) > 40°.

#### 2.2.2. Exclusion Criteria

- Orthopedic malformations or neurosurgical diseases;
- Disc degeneration or other conditions that may affect the interpretation of results (severe fibromyalgia and rheumatoid arthritis, in combination with other treatments);
- Surgery or pregnancy within 30 d;
- Breastfeeding;
- Wearing a hyperlordosis treatment device;
- Participation in similar studies.

### 2.3. Sample Size

In previous studies, a reduction in the LLA resulted in an effect size of d = 0.92 [19]. To achieve this effect size, calculations were made using G*power 3.1 software (developed by Franz Faul at University of Kiel, Kiel, Germany), setting α at 0.05 and β at 0.20. These calculations indicated that 20 participants were required in each group. To accommodate the dropout rate, an additional 20% of participants were included. Therefore, 48 participants were included.

### 2.4. Randomization and Blinding

The participants were randomly allocated to two groups using Random Allocation Software for Windows ver. 2.0, developed by Isfahan University, Iran. One group was assigned to perform abdominal exercises using the sprinter pattern (*n* = 20), whereas the other was assigned to perform the crunch exercises (*n* = 20). This study was conducted as a single-blind intervention; until the commencement of the exercise, participants remained unaware of the assigned exercises for both their own group and the other group. In addition, the radiographer was kept unaware of the assignment of exercises to specific patients. Both researchers and assessors were informed about the interventions during the evaluation phases, including the pre- and post-tests.

### 2.5. Intervention

Both groups initially underwent a pre-test in which the root of the mean square reference values was assessed using electromyography (EMG), and radiographic imaging was employed to measure the lordotic curve. Subsequently, the participants performed three sets of exercises according to their group: sprinter-pattern exercises (SPEs) or crunch exercises (CEs). Each set consisted of ten repetitions, with each movement held for 10 s. A 15 s rest interval was provided between sets to prevent muscle fatigue. The participants performed the intervention thrice. Abdominal muscle activity was measured using EMG during the exercise, and both groups underwent immediate post-exercise radiographic imaging for the post-test. To ensure accuracy during the study, the participants were instructed in advance by a trained physical therapist on the measurement methods and general procedure of the intervention program, and the same physical therapist performed the interventions throughout. Interventions were individually performed to ensure adequate attention and precision in the exercises. Furthermore, participants were closely monitored throughout the study for any adverse events.

#### 2.5.1. Sprinter-Pattern Exercise

**Figure 1:**
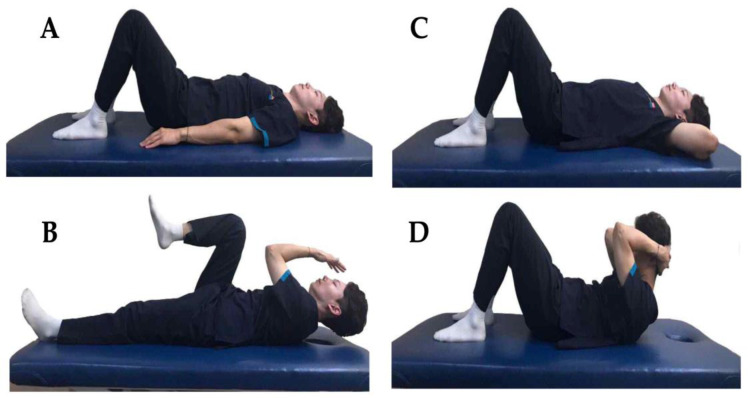
Interventions. (**A**) Position before the sprinter-pattern exercise, (**B**) sprinter-pattern exercise, (**C**) position before the crunch exercise, (**D**) crunch exercise.

For the SPE, the participants lay on their backs with their knees bent at a 45° angle and their arms resting beside them on the floor. Upon receiving a given signal, they raised their heads off the mat and simultaneously performed a series of coordinated arm and leg movements: flexing, adducting, and externally rotating one arm, while extending and internally rotating the corresponding leg. Conversely, they extended, abducted, and internally rotated the other arm while flexing, adducting, and externally rotating the opposing leg [14,25] (Figure 1A,B).

#### 2.5.2. Crunch Exercise

For the CE, the participants lay on their backs with their knees bent at a 45° angle and placed their hands behind their heads. When given the start signal, they lifted their heads and shoulders, elevating the inferior angle of the scapula from the mat [25] (Figure 1C,D).

### 2.6. Outcomes

The LLA and SHA were measured before and after the intervention, while abdominal muscle activity was measured during the intervention period.

#### 2.6.1. Lordotic Curve

The LLA and SHA were measured using an Accuray-525R diagnostic X-ray machine (Don Kang Medical System, Seoul, Republic of Korea). Radiographs were captured at distances of 2 m and 50 cm, with the following settings: exposure time, 0.8 s; tube voltage, 90 kVp; and tube current, 200 mA. Images were taken from the sagittal plane covering the pelvis to the lower spine while the subjects maintained an upright and functionally appropriate posture [26]. Throughout the study, a consistent and experienced radiologist from the hospital captured the radiographs. Although the clinical method for measuring the LLA has a decent reliability of 0.70–0.85 [27], radiographical methods have a higher reliability (0.87) [28]. Radiographic images were captured both before and after the intervention and subsequently underwent digitization before being transferred to a computer connected to the X-ray machine. The angles were then analyzed using built-in Viex Rex ver1.3.3.3.0 software (TechHeim, Seoul, Republic of Korea) on a vertical monitor with a resolution of 1080 × 1920.

1. Lumbar Lordotic Angle

**Figure 2:**
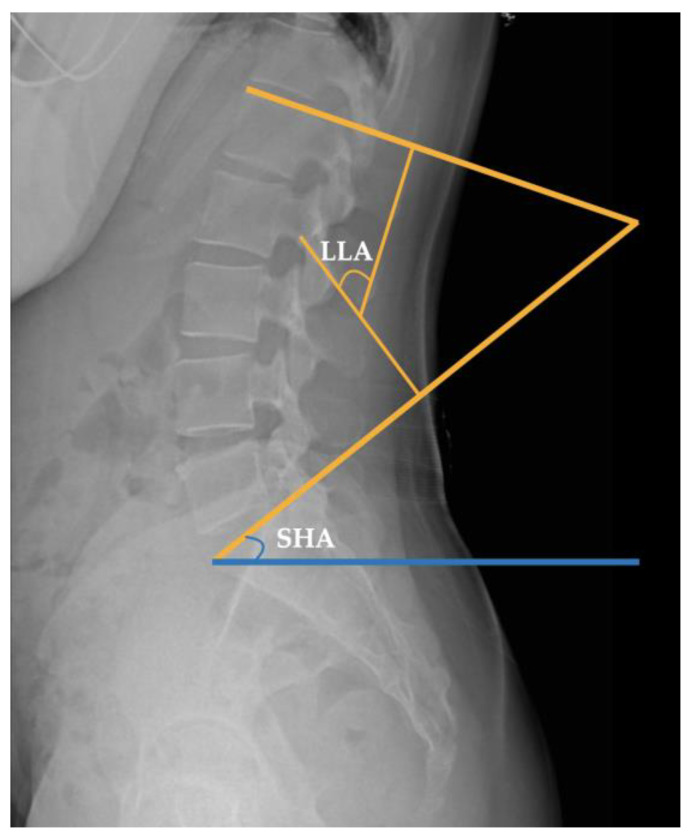
The lumbar lordotic angle (LLA) and sacrohorizontal angle (SHA).

The LLA measures the degree of lumbar curvature, which typically falls within a normal range of 40–50° [29]. This measurement is obtained by drawing lines along the upper plane of the L1 and S1 vertebrae, adding perpendicular lines to the converging side of the two, and subsequently measuring the angle of intersection [28]. This measurement technique is the prevailing standard in contemporary assessments of lumbar lordosis [30] (Figure 2).

2. Sacrohorizontal Angle

The SHA indicates the degree of sacral tilt with a normal angle of 40° [5]. There is a strong correlation between the SHA and lumbar lordosis (r = 0.84). An increase in the SHA typically results in an increase in lumbar lordosis [31,32]. This angle is determined by the alignment of a line parallel to the upper surface of the first sacral vertebra with the horizontal plane [33] (Figure 2).

#### 2.6.2. Abdominal Muscle Activity

Muscle activity was measured using surface EMG (Mini-wave Infinity Waterproof, Cometa Systems, Italy). The EMG signals were converted from analog to digital, and an EMG analysis software (Myoresearch XP Master ver. 1.07, Noraxon, Scottsdale, AZ, USA) was utilized, with the sampling rate set to 2000 Hz. The raw EMG data underwent full-wave rectification and were then quantified to an RMS of 150 ms. The data were filtered within a range of 20–450 Hz using a digital band-pass filter (Lancosh FIR). Ag/AgCl electrodes were used for the measurements. Before attaching the electrodes, to minimize skin resistance, any hair was removed, and the skin was exfoliated with sandpaper and cleaned with an alcohol swab. The specific attachment sites for each muscle are listed in Table 1 [34].

EMG was performed on the RA, EO, and IO muscles. To determine the baseline values for these muscles, the participants were asked to lie down with their knees bent and to lift both feet approximately 8 cm off the ground for 3 s. The average effective value was then calculated [35]. This action was used as a standard and normalized for comparison (% reference voluntary contraction (%RVC)).

Abdominal muscle activity was measured using EMG during the intervention. The average EMG signals were derived from the muscle activity measured during the assigned exercises, excluding the first and last second of each set. The effective values of each muscle recorded during the exercise motion were compared with the contraction values of the standard posture for analysis.

### 2.7. Data Analysis

Statistical analyses of the collected data were performed using SPSS Statistics 22 (IBM, New York, NY, USA). All participants underwent the Shapiro–Wilk normality test, and all variables were confirmed to follow a normal distribution. Descriptive statistics were used to assess participants’ general characteristics, while homogeneity tests were conducted using the chi-square test and the independent samples t-test. To compare the LLA, SHA, and muscle activity between the groups, an independent samples t-test was used. Moreover, to compare pre- and post-intervention within the groups, a paired sample t-test was used. The statistical significance level (α) for all data was set at 0.05. The interpretation based on the effect size utilized Cohen’s d, where 0.2, 0.5 and 0.8 indicates a small, medium, and large effect, respectively [36].

## 3. Results

### 3.1. General Characteristics

**Figure 3:**
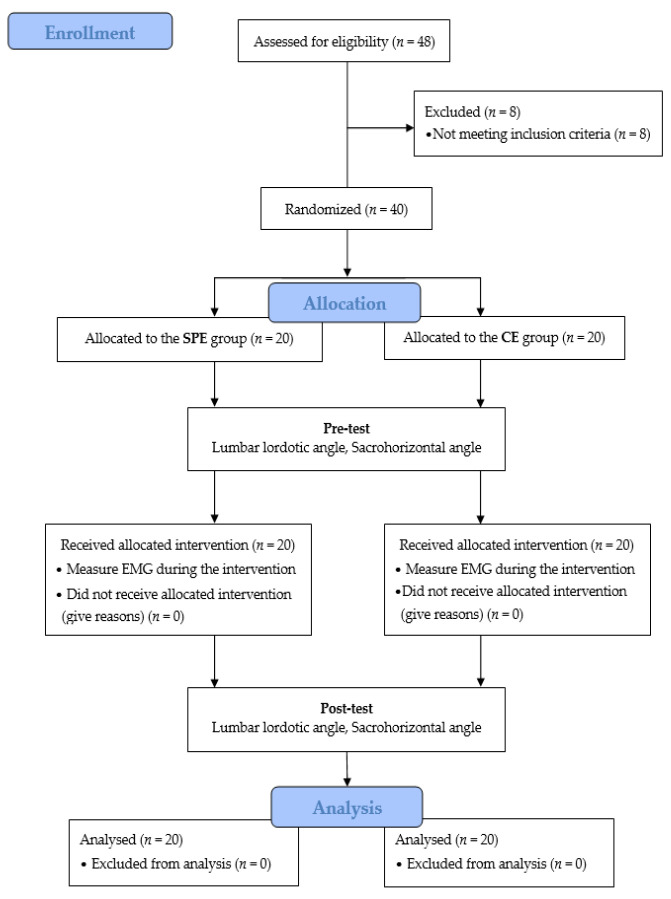
CONSORT (Consolidated Standards for Reporting of Trials) study flow.

Based on the study recruitment documents, a total of 40 individuals who voluntarily expressed interest and met the inclusion and exclusion criteria participated in the study (Figure 3). No significant differences were observed in the general characteristics of the enrolled participants between the groups (*p* > 0.05) (Table 2).

### 3.2. Changes in the Lumbar Lordotic Angle by Exercise Method

The changes in the LLA between the groups according to the exercise method are shown in Table 3 and Figure 3. Both the SPE and CE groups showed significant immediate post-intervention changes (*p* < 0.001). The difference in change from pre- and post-intervention showed a more positive improvement in the SPE group, which decreased by 4.62° compared to the CE group, which decreased by 2.24° (*p* < 0.001). Significant differences were found between the groups post-intervention, with the SPE group showing a statistically significant decrease compared to the CE group (*p* < 0.001).

### 3.3. Changes in Sacrohorizontal Angle by Exercise Method

The changes in the SHA between the groups according to the exercise method are shown in Table 3 and Figure 3. Both the SPE and CE groups showed significant immediate post-intervention changes (*p* < 0.001). The difference in change from pre- and post-intervention showed a more positive improvement in the SPE group, which decreased by 3.30°, compared to the CE group, which decreased by 1.81° (*p* < 0.001). Significant differences were observed between the groups post-intervention, and the SPE group showed a statistically significant decrease compared to the CE group (*p* < 0.01).

### 3.4. Changes in Muscle Activity by Exercise Method

**Figure 4:**
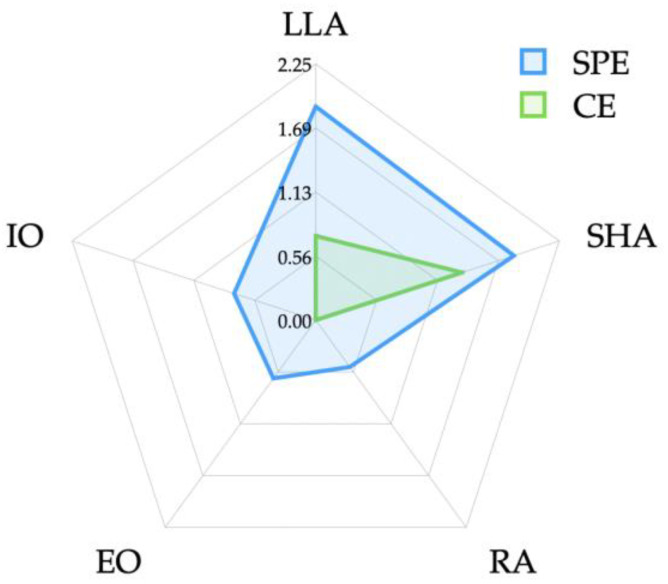
Effect size by exercise method. CE: crunch exercise, EO: external oblique, IO: internal oblique, LLA: lumbar lordotic angle, RA: rectus abdominis, SHA: sacrohorizontal angle, SPE: sprinter-pattern exercise.

The differences in the muscle activity between the groups are based on the exercise methods and are shown in Table 3 and Figure 4. No significant differences were observed in the muscle activity of the RA (*p* > 0.05). However, significant differences were observed in the EO and IO, with a significant increase in muscle activity observed in both muscles within the SPE group (*p* < 0.05).

## 4. Discussion

In this study, we examined the immediate effects of the SPE and CE on patients with hyperlordosis. Based on the research results, both LLA and SHA significantly decreased in both groups after the completion of the interventions (*p* < 0.001). These findings align with previous research by Olmedo-Buenrostro et al. [37], who reported that interventions, including Williams’ trunk-bending exercises, led to a reduction in both LLA and SHA. Similarly, Balasubramaniyam et al. [38] observed a decrease in LLA in participants with excessive lumbar lordosis after performing abdominal strengthening exercises. Therefore, increasing abdominal muscle strength is critical to treating hyperlordosis, which occurs when the pelvis tilts forward due to weakened abdominal muscles [7].

The present study emphasized the significance of abdominal exercises in reducing lumbar lordosis [5,29]. The RA, EO, and IO muscles, attached to the pelvis and ribs, contribute to trunk flexion through co-contraction, while trunk rotation is achieved through the co-contraction of the EO and IO muscles [5,39,40]. The abdominal muscles can influence lumbar lordosis by altering the position of the pelvis, and strong abdominal muscles can induce a posterior tilt of the pelvis, potentially reducing lumbar lordosis [41,42,43,44].

In this study, it was suggested that both the SPE and the CE may contribute to the reduction of LLA and SHA by increasing abdominal strength. According to the results, both groups closely reached the normal ranges of 40–50° for LLA and 40° for SHA [5,29]. Specifically, the SPE group showed a greater decrease, with LLA and SHA decreasing by 4.62° and 3.30°, respectively, compared to the CE group, where LLA and SHA decreased by 2.24° and 1.51°, respectively. This is thought to be because the SPE increases the activity of the EO and IO through the additional rotational resistance of trunk flexion and simultaneous crossed arm and leg movements, in coordination with the RA, whereas the CE primarily activates the RA over the EO and IO [45,46]. This indicates a significantly stronger effect of the SPE, suggesting the potential superiority of this specific exercise program in improving hyperlordosis. The critical findings of this study underscore the importance of the SPE in reducing hyperlordosis.

While this study provides evidence that both the SPE and the CE increase the activation of the abdominal muscles, the SPE group showed significantly higher activity in the EO and IO muscles, resulting in a more significant contribution to the reduction of LLA and SHA compared to the CE group (*p* < 0.05). However, the activity of the RA muscle was higher in the CE group compared to the SPE group, although there was no statistically significant difference (*p* > 0.05). The finding of higher activation shown in the EO and IO muscles in the SPE group, in comparison to the CE group, aligns with previous research indicating that abdominal exercises involving the arms and legs result in increased muscle activity in the EO and IO muscles [47,48,49]. This is likely due to the increased activity in the EO and IO muscles that occurs with a greater magnitude when movements from additional arm and leg exercises are applied to the trunk [50,51,52]. Therefore, abdominal exercises showing high muscle activity in these two muscles are suggested to be suitable exercises for individuals with low back pain [53]. Also, this multi-faceted muscle engagement is instrumental in fostering spinal movement and trunk stabilization, contributing to enhanced spinal and pelvic stability [47,54].

Carolyn Richardson et al. [55] suggested that abdominal exercises resisting trunk rotation to maintain a neutral spinal position are suitable for core stabilization exercises. These exercises minimize the contraction of the RA while inducing cooperative contractions of the IO and EO, making them suitable for core stabilization exercises [55]. In contrast, conventional abdominal exercises that involve excessive flexion and extension of the spine, such as sit-ups, which activate the abdominal muscles, increase the risk of low back pain by generating significant shear force and compressive force on the intervertebral discs and lumbar vertebrae [56,57]. Our research findings indicate the positive effect of the SPE in reducing lumbar lordosis by strengthening the abdominal muscles. Therefore, the SPE could be considered as a beneficial and targeted intervention for individuals dealing with lumbar lordosis.

While this study provides valuable insight into the immediate effects of the SPE and CE on lumbar lordosis and abdominal muscle activity, it is important to recognize certain limitations. The immediate post-intervention measurements captured in this study, while informative, do not necessarily reflect the potential for long-term effects or sustained relief of back pain. As suggested by McPherson and Watson [58], short-term interventions may not guarantee long-term benefits. Therefore, future research efforts should prioritize the investigation of the sustained effects of these exercises, including assessments of back pain severity and comparisons with other commonly used abdominal exercises. In addition, it is crucial to recognize the inherent individual variability in response to these exercises. Thus, future research should take a more personalized approach and tailor exercise programs to individual needs. There is also a need to assess the adaptability and responsiveness of different demographic groups to these interventions. Therefore, it is imperative to include a diverse pool of participants and to account for individual baseline characteristics. Moreover, exploring the underlying mechanisms by which these exercises contribute to muscle activity and spinal alignment holds promise for informing therapeutic interventions. Understanding these mechanisms may contribute to more tailored exercise recommendations for individuals with hyperlordosis. This comprehensive approach may lead to a more nuanced understanding of how these exercises work in different populations. It will allow researchers to formulate personalized therapeutic strategies that address the unique needs of individuals, contributing to a more effective and targeted approach to the treatment of hyperlordosis. This expanded knowledge base would not only improve the effectiveness of interventions but also contribute to a broader understanding of lumbar spine health.

## 5. Conclusions

In conclusion, this study provides valuable insight into the immediate effects of the SPE and CE on lumbar lordosis and abdominal muscle activity in individuals with hyperlordosis. The SPE and CE have the potential to strengthen the abdominal muscles and consequently reduce lumbar lordosis in patients with hyperlordosis. In particular, the SPE shows superior efficacy in stimulating abdominal muscles and reducing lumbar lordosis compared to the CE. Therefore, the SPE might be an ideal option for the intervention in patients with hyperlordosis.

## Figures and Tables

**Table 1:** Electromyography attachment sites.

Abdominal Muscles	Attachment Site
Rectus abdominis	A point approximately 2 cm lateral to and above the umbilicus, parallel to the muscle fibers.
External oblique	A point above the anterior superior iliac spine on the lateral surface of the rectus abdominis.
Internal oblique	The midpoint between the border of the inguinal ligament and outer corner of the rectus abdominis and the triangle formed by the anterior superior iliac spine and the umbilicus.

**Table 2:** General characteristics of participants.

	SPE (*n* = 20)	CE (*n* = 20)	*p*
Sex (male/female)	9/11	11/9	0.539
Age (years)	34.40 ± 7.18	34.25 ± 6.96	0.413
Height (cm)	168.80 ± 7.19	169.40 ± 7.37	0.625
Weight (kg)	61.35 ± 10.00	61.97 ± 9.11	0.839
Body mass index (kg/m^2^)	21.34 ± 1.75	21.32 ± 1.42	0.968

Values are presented as mean ± standard deviation. CE: crunch exercise, SPE: sprinter-pattern exercise.

**Table 3:** Changes in each variable depending on the exercise method.

Variables	SPE (*n* = 20)	CE (*n* = 20)	Between-Group Differences ^‡^
Lumbar lordotic angle (°)			
Baseline (A)	56.24 ± 2.57	55.49 ± 3.11	
Immediate (B)	51.63 ± 2.21	53.25 ± 2.79	−1.62 [−3.02, −0.22] ^†^
Change from A to B	4.62 ± 1.31	2.24 ± 1.18	
Change from A to B ^‡^	1.876 [4.05, 5.19] ***	0.739 [1.72, 2.76] ***	1.851 [1.58, 3.18] ***
Sacrohorizontal angle (°)			
Baseline (A)	42.78 ± 1.50	41.98 ± 1.18	
Immediate (B)	39.48 ± 1.97	40.17 ± 1.42	−0.69 [−1.68, 0.30] ^†^
Change from A to B	3.30 ± 1.60	1.81 ± 1.17	
Change from A to B ^‡^	1.831 [2.60, 4.01] ***	1.352 [1.30, 2.32] ***	1.031 [0.59, 2.40] **
Muscle activation (%)			
Rectus abdominis	155.90 ± 38.17	179.03 ± 49.71	−23.12 [−48.47, 2.23] ^†^
External oblique	75.96 ± 21.00	62.90 ± 17.20	13.06 [1.44, 24.68] ^†,^*
Internal oblique	165.76 ± 46.96	132.08 ± 39.87	33.68 [4.34, 63.02] ^†,^*

Values are presented as mean ± standard deviation. CE, crunch exercise; SPE, sprinter-pattern exercise. ^†^ Mean change (95% confidence interval). ^‡^ Cohen’s d (95% confidence interval). * *p* < 0.05, ** *p* < 0.01, *** *p* < 0.001.

## Data Availability

The data presented in this study are available on request from the corresponding author.
